# Intronic regulation of *Aire* expression by Jmjd6 for self-tolerance induction in the thymus

**DOI:** 10.1038/ncomms9820

**Published:** 2015-11-04

**Authors:** Toyoshi Yanagihara, Fumiyuki Sanematsu, Tetsuya Sato, Takehito Uruno, Xuefeng Duan, Takahiro Tomino, Yosuke Harada, Mayuki Watanabe, Yuqing Wang, Yoshihiko Tanaka, Yoichi Nakanishi, Mikita Suyama, Fukui Yoshinori

**Affiliations:** 1 Division of Immunogenetics, Department of Immunobiology and Neuroscience, Medical Institute of Bioregulation, Kyushu University, 3-1-1 Maidashi, Higashi-ku, Fukuoka 812-8582, Japan; 2 Research Institute for Diseases of the Chest, Graduate School of Medical Sciences, Kyushu University, 3-1-1 Maidashi, Higashi-ku, Fukuoka 812-8582, Japan; 3 Research Centre for Advanced Immunology, Kyushu University, 3-1-1 Maidashi, Higashi-ku, Fukuoka 812-8582, Japan; 4 Division of Bioinformatics, Multi-scale Research Centre for Medical Science, Medical Institute of Bioregulation, Kyushu University, 3-1-1 Maidashi, Higashi-ku, Fukuoka 812-8582, Japan; 5 Section of Infection Biology, Department of Functional Bioscience, Fukuoka Dental College, 2-15-1 Tamura, Sawara-ku, Fukuoka 814-0175, Japan

## Abstract

The thymus has spatially distinct microenvironments, the cortex and the medulla, where the developing T-cells are selected to mature or die through the interaction with thymic stromal cells. To establish the immunological self in the thymus, medullary thymic epithelial cells (mTECs) express diverse sets of tissue-specific self-antigens (TSAs). This ectopic expression of TSAs largely depends on the transcriptional regulator Aire, yet the mechanism controlling Aire expression itself remains unknown. Here, we show that Jmjd6, a dioxygenase that catalyses lysyl hydroxylation of splicing regulatory proteins, is critical for Aire expression. Although Jmjd6 deficiency does not affect abundance of *Aire* transcript, the intron 2 of *Aire* gene is not effectively spliced out in the absence of Jmjd6, resulting in marked reduction of mature Aire protein in mTECs and spontaneous development of multi-organ autoimmunity in mice. These results highlight the importance of intronic regulation in controlling Aire protein expression.

## Abstract (web summary)

Jmjd6 is a dioxygenase that catalyses lysyl hydroxylation of splicing regulatory proteins. Here the authors show that Jmjd6 directs splicing of a central tolerance regulator Aire in medullary thymic epithelial cells, and that Jmjd6 deficiency leads to loss of Aire and multi-organ autoimmunity in mice.

## Graphical abstract

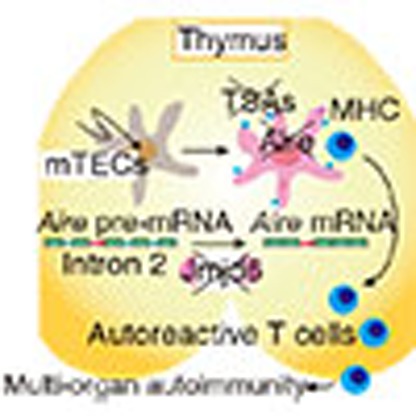

The thymus has spatially distinct microenvironments, the cortex and the medulla, where developing T-cells are selected to mature or die through the interaction with thymic stromal cells^1^,^2^. Cortical thymic epithelial cells (cTECs), a major stromal cell-type in the cortex, direct differentiation of CD4^+^CD8^+^ immature thymocytes that are capable of recognizing self-major histocompatibility complex (MHC) molecules. On the other hand, medullary thymic epithelial cells (mTECs) play an important role in self-tolerance induction by eliminating self-reactive T-cells. A unique property of mTECs is their expression of diverse sets of peripheral tissue-specific self-antigens (TSAs)^3^,^4^. This ectopic expression of TSAs largely depends on the transcriptional regulator Aire^5^,^6^,^7^,^8^, which is expressed in mature mTECs^9^,^10^,^11^. The homozygous mutations of human AIRE cause an autoimmune disease known as autoimmune-polyendocrinopathy-candidiasis ectodermal dystrophy^12^,^13^. Similarly, Aire-deficient mice develop multi-organ autoimmunity with the failure to delete self-reactive T-cells^5^,^14^,^15^. Despite an important role in self-tolerance induction, the mechanism controlling Aire expression itself is poorly understood.

Alternative splicing is a major cellular mechanism in metazoans for generating proteomic diversity^16^,^17^. This is a posttranscriptional process in which premature transcripts are selectively cut and joined in more than one way to generate multiple mRNAs from a single gene. There are three forms of alternative splicing: exon skipping, alternative splice site usage and intron retention^17^. Of these, intron retention is the least frequent alternative splicing form^17^, which occurs when an intron, having been transcribed as a part of a pre-mRNA, is not spliced out. The sequence structure of most introns consists of a short 5′ splice site boundary, a minimal AG dinucleotide 3′ splice site boundary, a catalytic adenosine and a polypyrimidine tract (PPT)^16^. Mechanistically, intron retention is considered to be the result of weak splice site sequences that are not properly recognized by spliceosome^18^. Intron retention often inserts a premature termination codon in the mature transcript that would then be degraded by non-sense-mediated decay^19^. Therefore, its physiological significance has been so far overlooked.

Jmjd6 is a member of the JmjC-domain containing proteins that are involved in a wide range of oxidation reactions^20^. Jmjd6 was initially identified as a phosphatidylserine receptor that mediates recognition and engulfment of apoptotic cells^21^. However, recent evidence indicates that Jmjd6 is a nuclear protein and catalyses lysyl hydroxylation of multiple substrates, including splicing regulatory proteins, transcription factors and histones, in a manner dependent on the Fe(II) and 2-oxoglutarate^22^,^23^,^24^,^25^. Jmjd6 deficiency in mice causes abnormal development of multiple organs during embryogenesis and led to perinatal lethality^26^,^27^,^28^; yet, its role in the immune system and immune responses remain unclear. Here we show that Jmjd6 plays a key role in induction of central tolerance by controlling Aire expression in mTECs. Although Jmjd6 deficiency did not affect abundance of *Aire* transcript, the intron 2 of *Aire* gene was not effectively spliced out in the absence of Jmjd6, owing to the unique 3′ splice site sequence. As a result, the expression of Aire protein was markedly reduced in Jmjd6-deficient (*Jmjd6*^*−/−*^) mTECs, and T-cells generated in such thymic microenvironments caused multi-organ autoimmunity in mice. Our findings indicate that Aire protein expression is tightly controlled through two discrete steps, intron retention and its relief, the latter of which involves the enzymatic activity of Jmjd6.

## Results

### Impaired tolerance induction by *Jmjd6*^*−/−*^ thymic stroma

**Figure 1:**
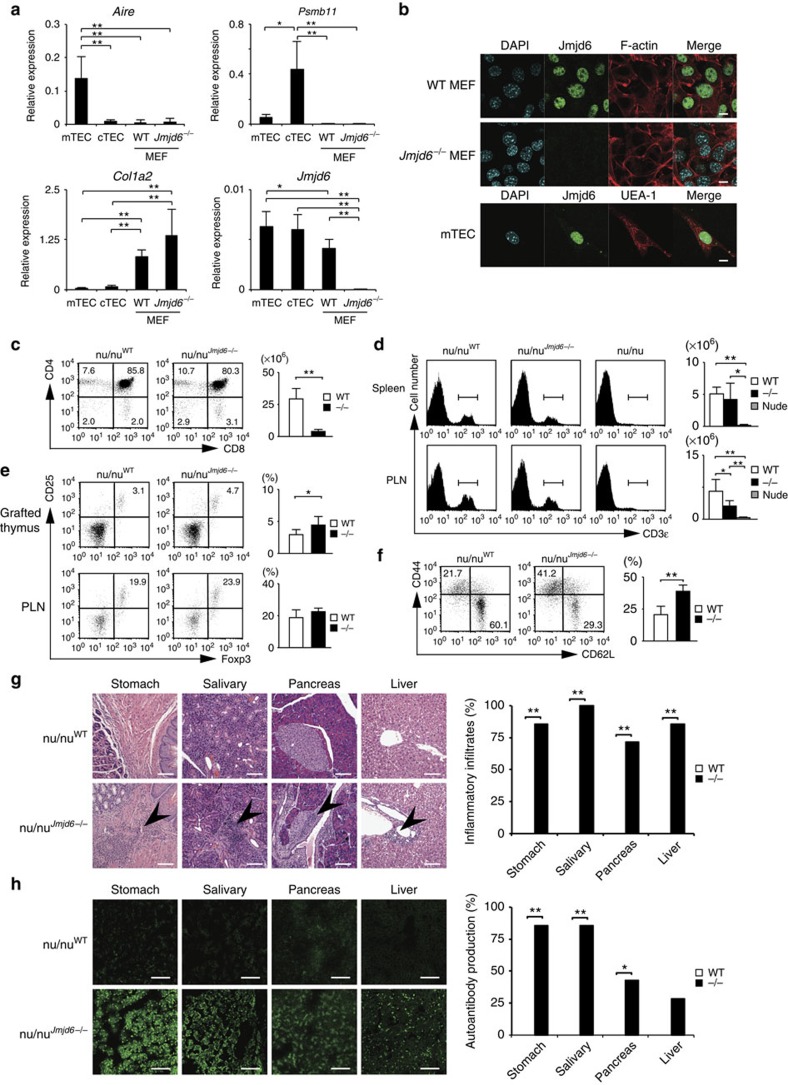
Jmjd6 expression in thymic stroma is required for T-cell tolerance induction. (**a**) Real-time PCR analyses for *Jmjd6* expression in mTECs, cTECs and MEFs. (**b**) Immunofluorescence staining showing nuclear localization of Jmjd6. UEA-1 was used to detect mTECs. Data are representative of two independent experiments. (**c**) Expression of CD4 and CD8 on thymocytes differentiated in the grafts. The number of total thymocytes was compared between nu/nu^WT^ (*n*=8) and nu/nu^*Jmjd6−/−*^ mice (*n*=7). (**d**) The numbers of CD3ɛ^+^ T-cells in the spleen and PLNs were compared among nu/nu^WT^ (*n*=8), nu/nu^*Jmjd6−/−*^ (*n*=7) and nude (*n*=3) mice. (**e**) The numbers of of CD25^+^Foxp3^+^ regulatory T-cells in the grafted thymi and PLNs were compared between nu/nu^WT^ (*n*=8) and nu/nu^*Jmjd6−/−*^ mice (*n*=7). (**f**) Expression of CD44 and CD62L on PLN CD4^+^ T-cells from nu/nu^WT^ (*n*=8) and nu/nu^*Jmjd6−/−*^ mice (*n*=7). (**g**) Haematoxylin and eosin staining of tissues from nu/nu^WT^ and nu/nu^*Jmjd6−/−*^ mice. Scale bars, 100 μM. The percentage of each organ with inflammatory infiltrates was compared between nu/nu^WT^ (*n*=8) and nu/nu^*Jmjd6−/−*^ (*n*=7) mice. (**h**) Immunofluorescence staining showing the presence of autoantibodies (green) in nu/nu^*Jmjd6−/−*^ mice. Scale bars, 100 μM. The percentage of mice with autoantibodies against each tissue was compared between nu/nu^WT^ (*n*=8) and nu/nu^*Jmjd6−/−*^ mice (*n*=7). Data are collected from four (**a**), six (**c**–**g**) and three (**h**) separate experiments and are expressed as mean±s.d. *P<0.05; **P<0.01 (two-tailed Student's *t*-test).

A previous study has shown that intrathymic T-cell development is partially impaired in *Jmjd6*^*−/−*^ embryos^27^. However, *Jmjd6*^*−/−*^ T-cells developed normally in chimeric mice reconstituted with fetal liver cells from the knockout mice (Supplementary Fig. 1), suggesting that Jmjd6 may play a role in thymic stromal cells. Indeed, Jmjd6 was expressed in mTECs and cTECs as well mouse embryonic fibroblasts (MEFs) (Fig. 1a), and its nuclear localization was confirmed by immunofluorescent staining (Fig. 1b). To assess a possible role of Jmjd6 in TECs, we prepared fetal thymi from wild-type (WT) and *Jmjd6*^*−/−*^ embryos at embryonic days 15.5 (E15.5) and grafted them under the renal capsule of athymic C57BL/6 nude mice (B6.Cg-Foxn1<nu>/Nrs) (designated nu/nu^WT^ and nu/nu^*Jmjd6−/−*^) after depleting haematopoietic cells by incubation in 2′-deoxyguanosine (2-DG). Although the size of the thymi developed in nu/nu^*Jmjd6−/−*^ mice was apparently small, their CD4^+^CD8^+^ thymocytes normally differentiated into CD4^+^CD8^–^ or CD4^–^CD8^+^ mature T-cells (Fig. 1c), indicating that *Jmjd6*^*−/−*^ cTECs are functionally intact to support positive selection of CD4^+^CD8^+^ immature thymocytes. Indeed, nu/nu^WT^ and nu/nu^*Jmjd6−/−*^ mice had almost comparable numbers of mature T-cells in the spleen and peripheral lymph nodes (PLNs) 10 weeks after grafting (Fig. 1d). Jmjd6 deficiency in thymic stroma did not impair generation of regulatory T-cells (Fig. 1e). However, the frequency of CD4^+^ T-cells with an activated/memory phenotype (CD44^hi^CD62L^lo^) significantly increased in nu/nu^*Jmjd6−/−*^ mice (Fig. 1f). Histological examination demonstrated inflammatory infiltrates in the stomach, salivary gland, pancreas and liver of nu/nu^*Jmjd6−/−*^ mice (Fig. 1g). In addition, nu/nu^*Jmjd6−/−*^ mice, but not nu/nu^WT^ mice, generated autoantibodies against these tissues (Fig. 1h). Thus, Jmjd6 expression in thymic stroma is required for induction of self-tolerance in developing T-cells.

### Defective Aire expression in *Jmjd6*^*−/−*^ mTECs

**Figure 2:**
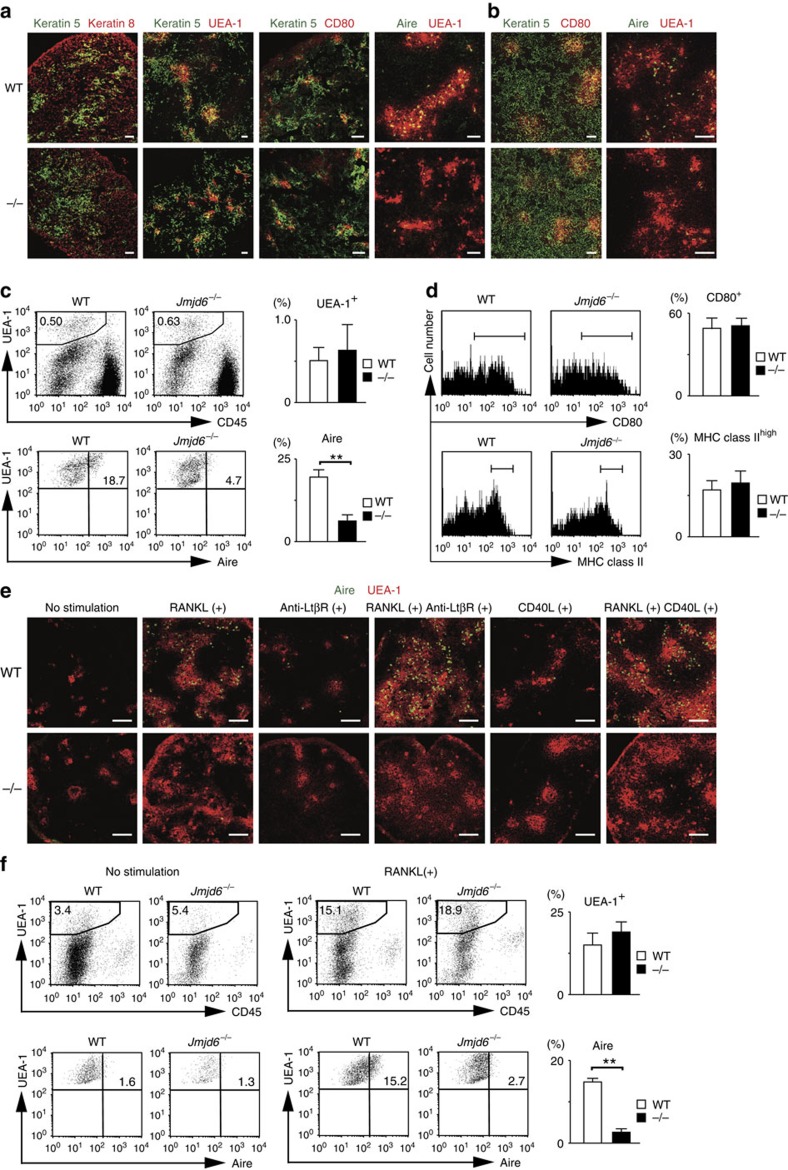
Defective Aire protein expression in *Jmjd6*^*−/−*^ mTECs. (**a**) Thymic sections from E18.5 WT and *Jmjd6*^*−/−*^ embryos were stained with UEA-1 and/or antibodies specific for keratin 5, keratin 8, CD80 and Aire. Scale bars, 50 μM. (**b**) Immunofluorescence analysis of thymi from WT and *Jmjd6*^*−/−*^ embryos grafted under the kidney capsule of C57BL/6 mice. Six weeks after engraftment, thymic sections were stained with UEA-1 and/or antibodies specific for keratin 5, CD80 and Aire. Scale bars, 50 μM. (**c**) Flow cytometric analyses for Aire expression in mTECs prepared from E18.5 embryos. The percentages of UEA-1^+^ mTECs and Aire^+^ UEA-1^+^ mTECs were compared between WT (*n*=3) and *Jmjd6*^*−/−*^ mice (*n*=5). (**d**) Flow cytometric analyses for expression of CD80 and MHC class II in mTECs prepared from E18.5 embryos. The percentages of CD80^+^ mTECs and MHC class II^high^ mTECs were compared between WT (*n*=3) and *Jmjd6*^*−/−*^ mice (*n*=4). (**e**) Following stimulation with RANKL, anti-LtβR antibody and/or CD40L for 4 days, 2-DG-treated fetal thymic stroma from WT and *Jmjd6*^*−/−*^ embryos were stained with UEA-1 and anti-Aire antibody. Scale bars, 50 μM. (**f**) Flow cytometric analyses for Aire expression in mTECs prepared from FTOC with or without RANKL stimulation. The percentages of UEA-1^+^ mTECs and Aire^+^ UEA-1^+^ mTECs were compared between RANKL-stimulated WT (*n*=3) and *Jmjd6*^*−/−*^ samples (*n*=3). Data are collected from three (**c**,**d**,**f**) separate experiments and are expressed as mean±s.d. **P<0.01 (two-tailed Student's *t*-test).

To examine the function of Jmjd6 in TECs in more detail, we performed histological analyses. Haematoxylin and eosin staining of E18.5 thymi revealed that both the cortex and the medulla poorly developed in the absence of Jmjd6 (Supplementary Fig. 2). However, no significant difference in thymic architecture was found between WT and *Jmjd6*^*−/−*^ mice when thymic sections were stained for the cTEC marker keratin 8 and mTEC marker keratin 5 (ref. 29) (Fig. 2a). In addition, mTECs of both WT and *Jmjd6*^*−/−*^ embryos were comparably stained with *Ulex europaeus* agglutinin-1 (UEA-1), a lectin that binds to mature mTECs^29^ (Fig. 2a), suggesting that Jmjd6 deficiency does not affect mTEC maturation. Consistent with this, the expression of another maturation marker CD80 was unchanged between WT and *Jmjd6*^*−/−*^ mTECs^4^,^6^,^30^ (Fig. 2a). Surprisingly, however, the expression of Aire in mTECs of *Jmjd6*^*−/−*^ embryos was markedly reduced at E18.5, as compared with that in WT controls (Fig. 2a and Supplementary Fig. 3a). This does not simply reflect delayed Aire expression in *Jmjd6*^*−/−*^ thymi, because similar results were obtained when the ‘arteficial' thymi from *Jmjd6*^*−/−*^ embryos were analysed 6 weeks after grafting (Fig. 2b and Supplementary Fig. 3b).

To determine more precisely the expression level of Aire in mTECs, we performed flow cytometric analyses. E18.5 thymi from both WT and *Jmjd6*^*−/−*^ mice contained comparable numbers of CD45^–^UEA-1^+^ mTECs (Fig. 2c). Although cell-surface expressions of CD80 and MHC class II were unchanged between WT and *Jmjd6*^*−/−*^ mTECs (Fig. 2d), intracellular staining of Aire revealed that the proportion of Aire^+^ mTECs in *Jmjd6*^*−/−*^ thymi was reduced to 25.1% of the WT level (Fig. 2c). Thus, Jmjd6 deficiency reduces Aire protein expression in mTECs without affecting mTEC maturation.

The Aire expression is induced in mTECs through interaction with cells such as lymphoid tissue inducers, positively selected TCRαβ^+^ thymocytes, and TCR Vγ5^+^ dendritic epidermal T-cell progenitors^30^,^31^,^32^,^33^, which is mainly mediated by the signals through tumour necrosis factor receptor family members, such as receptor activator of NF-κB (RANK), CD40 and lymphotoxin β receptor (LtβR)^34^,^35^,^36^. When 2-DG-treated WT fetal thymic stroma was stimulated *in vitro* with RANK-ligand (RANKL), the Aire expression was markedly induced in a subset of UEA-1^+^ mTECs (Fig. 2e and Supplementary Fig. 3c). Although CD40-ligand (CD40L) or anti-LtβR antibody alone was not effective, they synergistically act with RANKL to augment Aire expression in WT mTECs (Fig. 2e and Supplementary Fig. 3c). However, the Aire expression was hardly detected in *Jmjd6*^*−/−*^ mTECs even after stimulation with RANKL in combination with CD40L or anti-LtβR antibody (Fig. 2e and Supplementary Fig. 3c). This was again confirmed by flow cytometric analyses (Fig. 2f). These results indicate that Jmjd6 is critical for Aire expression in mTECs.

### Intronic regulation of *Aire* gene by Jmjd6

**Figure 3:**
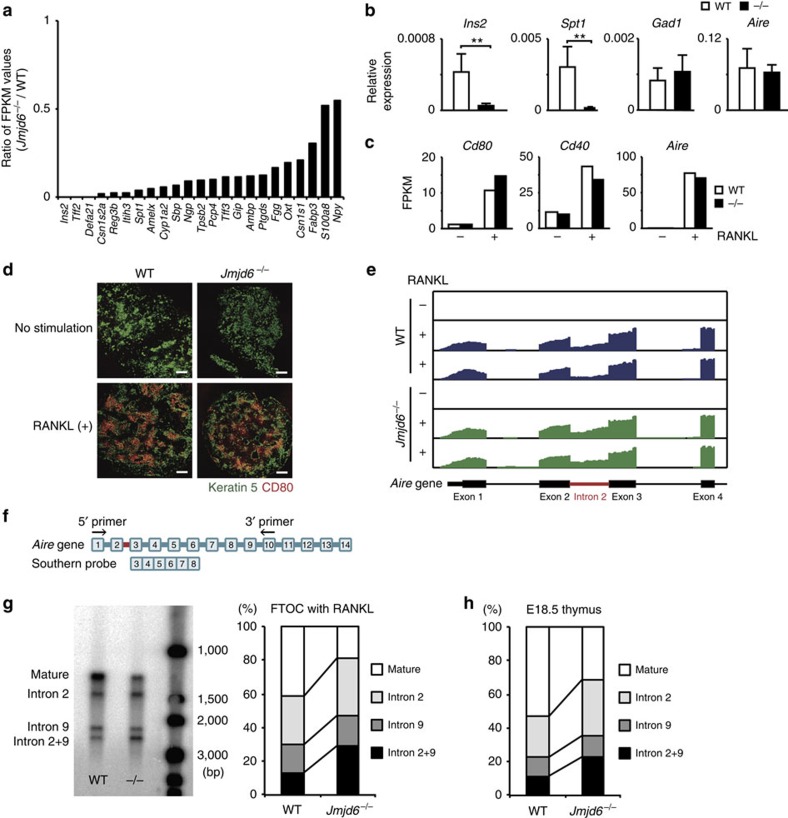
Jmjd6 controls Aire protein expression through intron retention. (**a**) Reduced expression of 23 Aire-dependent TSAs in *Jmjd6*^*–/–*^ thymic stroma. Data were obtained from RNAseq analyses for RANKL-stimulated samples. (**b**) Comparison of gene expression of Aire-dependent and -independent TSAs between RANKL-stimulated WT (*n*=5) and *Jmjd6*^*–/–*^ (*n*=5) thymic stroma by real-time PCR. *Ins2*, insulin 2; *Spt1*, salivary protein 1; *Gad1*, GAD67. Data are expressed as mean±s.d. **P<0.01 (two-tailed Student's *t*-test). (**c**) Comparable expression of *Cd80*, *Cd40* and *Aire* in WT and *Jmjd6*^*–/–*^ thymic stroma. Data were obtained from RNAseq analyses for RANKL-stimulated samples. (**d**) Following stimulation with RANKL for 4 days, 2-DG-treated fetal thymic stroma from WT and *Jmjd6*^*–/–*^ embryos were stained with antibodies specific for keratin 5 and CD80. Scale bars, 100 μM. Data are representative of three independent experiments. (**e**) RNA-seq read densities at the *Aire* gene locus. Data were obtained from WT and *Jmjd6*^*–/–*^ thymic stroma stimulated with (+) or without (–) RANKL. (**f**) Schematic representation of primers and a probe used for RT-PCR and Southern blotting. (**g**) RT-PCR and Southern blot analysis showing multiple *Aire* transcripts with or without retention of intron 2, intron 9, and intron 2 plus intron 9. The intensity of each band was compared between RANKL-stimulated WT and *Jmjd6*^*–/–*^ thymic stroma (*n*=3). (**h**) The frequency of each *Aire* transcript was compared between E18.5 WT (*n*=4) and *Jmjd6*^*–/–*^ (*n*=3) thymi, as in (**g**).

As Jmjd6 is a nuclear protein that catalyses lysyl hydroxylation of multiple substrates^22^,^23^,^24^,^25^, it seemed likely that Jmjd6 deficiency affects transcription of both upstream and downstream genes of *Aire*. To comprehensively identify genes controlled by Jmjd6, we prepared 2-DG-treated fetal thymic stroma with or without RANKL stimulation from WT and *Jmjd6*^*−/−*^ mice and analysed their transcriptomes by RNA sequencing (RNA-seq). Although the gene expression of *Jmjd6* was unchanged between before and after stimulation (Supplementary Fig. 4), 1,850 genes were induced in response to RANKL stimulation, among which 1,020 genes were expressed in WT fetal thymic stroma at significantly higher levels than those in *Jmjd6*^*−/−*^ samples (see top 200 genes in Supplementary Table 1). These included 23 genes encoding Aire-dependent TSAs such as insulin 2 and salivary protein 1 (refs 5, 6, 8) (Fig. 3a). This reduction of Aire-dependent TSAs was further confirmed by quantitative real-time PCR using samples from *Jmjd6*^*−/−*^ fetal thymic stroma and *Jmjd6*^*−/−*^ E18.5 thymi (Fig. 3b and Supplementary Fig. 5). On the other hand, Jmjd6 deficiency did not affect gene expression of Aire-independent TSA, glutamate decarboxylase 67 (GAD67)^5^,^6^,^8^ (Fig. 3b and Supplementary Fig. 5). Similarly, gene expression of CD80 and CD40 were unaffected in the absence of Jmjd6 (Fig. 3c). Consistent with this finding, immunohistochemical analysis revealed that RANKL-induced CD80 induction normally occurred even in *Jmjd6*^*−/−*^ mTECs (Fig. 3d). Unexpectedly, however, the abundance of *Aire* transcript was also comparable between RANKL-stimulated WT and *Jmjd6*^*−/−*^ samples (Fig. 3b,c), suggesting that Jmjd6 controls Aire expression through a posttranscriptional mechanism.

To explore the underlying mechanism, we next analysed pre-mRNA splicing. Among 84,708 introns of detected genes, 1,051 introns were selected, because they were expressed at a relatively high frequency (intronic FPKM>10). Bioinformatics analysis identified 57 introns preferentially expressed in *Jmjd6*^*−/−*^ thymic stroma under RANKL-stimulated condition (Supplementary Table 2). These included the intron 2 of *Aire*, which was found in RANKL-treated *Jmjd6*^*−/−*^ samples at 1.9 times higher frequency than that in the WT sample (Fig. 3e). This was further confirmed by RT-PCR followed by Southern blotting (Fig. 3f). Amplification of *Aire* cDNA with primers specific for the sequence of the exons 1 and 10 yielded four bands corresponding to the *Aire* transcript with or without retention of intron 2, intron 9 and intron 2 plus intron 9 (Fig. 3g). By measuring the intensity of each band, 41.5% of the *Aire* transcripts expressed in RANKL-stimulated WT fetal thymic stroma were found to be mature form, whereas this value decreased to 18.6% in *Jmjd6*^*−/−*^ samples (Fig. 3g), because of increase in the frequency of retention of intron 2 and/or intron 9. Similar results were obtained when E18.5 thymi of WT and *Jmjd6*^*−/−*^ embryos were analysed with this method (Fig. 3h).

**Figure 4:**
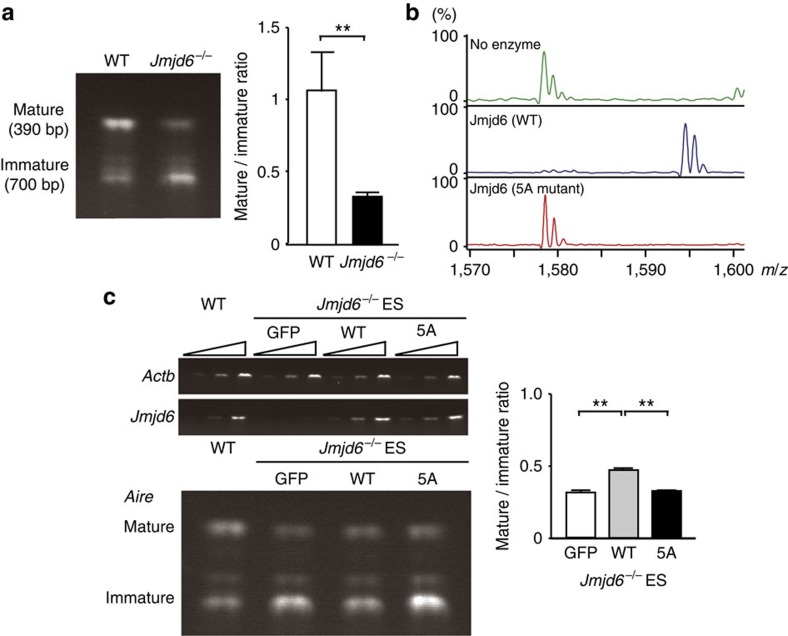
The enzymatic activity of Jmjd6 is required for intronic regulation of *Aire* gene. (**a**) Comparison of the ratio of mature (without intron 2) to immature (with intron 2) *Aire* transcript between WT and *Jmjd6*^*−/−*^ ES cells (*n*=4). Amplification of *Aire* cDNA was performed with primers specific for the sequence of the exons 1 and 3. (**b**) MALDI-TOF analyses for lysyl-hydroxylation of LUC7L2_267–278_ peptide. The mass corresponding to LUC7L2_267–278_ +16, equivalent to one hydroxylation, was identified in the presence of WT Jmjd6, but not 5A mutant. Data are representative of two independent experiments. (**c**) Effect of the 5A mutation on intron 2 retention of *Aire* gene. 30 h after transfection of *Jmjd6*^*−/−*^ ES cells with genes encoding GFP-fusion Jmjd6, GFP-fusion 5 A mutant or GFP alone, the ratio of mature to immature *Aire* transcript was analysed by RT-PCR (*n*=4). In (**a** and **c**), data are collected from four separate experiments and are expressed as mean±s.d. **P<0.01 (two-tailed Student's *t*-test).

It is known that Aire is also expressed in the reproductive organs and embryonic stem (ES) cells^8^,^37^,^38^. To examine whether a similar mechanism operates in ES cells, we developed *Jmjd6*^*−/−*^ ES cells and compared their *Aire* transcripts with those of WT ES cells. Jmjd6 deficiency in ES cells markedly increased the Aire transcript containing intron 2 (Fig. 4a), indicating that Aire expression in ES cells is also controlled by Jmjd6 through intron retention. Recent structural analysis of the catalytic domain of Jmjd6 indicated amino acid residues critical for binding to Fe(II), 2-oxoglutarate and substrate lysine^39^. When five of these amino acid residues were mutated to alanine (designated 5 A mutant), the catalytic activity of Jmjd6 to hydroxylate lysine residues was completely lost (Fig. 4b). Although the transient expression of WT Jmjd6 in *Jmjd6*^*−/−*^ ES cells significantly, albeit incompletely, improved the ratio of mature *Aire* transcript (without intron 2) to immature transcript (with intron 2), such improvement was not achieved by the 5 A mutant (Fig. 4c). These results suggest that Jmjd6 controls splicing events of *Aire* gene depending on its enzymatic activity.

### The nature of immature Aire protein

**Figure 5:**
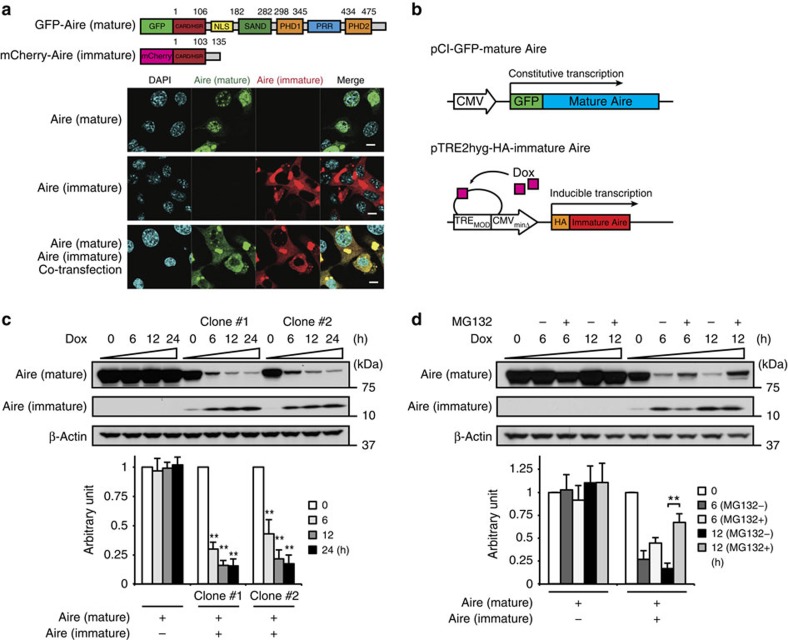
The effect of immature Aire protein on localization and stability of mature Aire protein. (**a**). Subcellular localization of GFP-tagged mature Aire protein and mCherry-tagged immature Aire protein generated by intron 2 retention. Data are representative of two independent experiments. (**b**) Schematic representation of pCI-GFP-mature Aire and pTRE2hyg-HA-immature Aire. (**c**,**d**) After treatment with doxycycline (Dox) for the indicated times in the presence or absence of MG132, expressions of mature and immature Aire proteins were analysed for HEK293 cells stably expressing both pCI-GFP-mature Aire and pTRE2hyg-HA-immature Aire or pTRE2hyg. Data (mean±s.d., *n*=3) are expressed as the level of mature Aire protein after normalization of the 0 h value to an arbitrary value of 1. **P<0.01 (two-tailed Student's *t*-test).

The retention of intron 2 results in an appearance of a premature termination codon at the *N*-terminal portion of Aire (Fig. 5a and Supplementary Fig. 6), leaving only 103 amino acid residues presumably intact. To know the nature of this immature Aire protein generated by intron 2 retention, we first analysed its subcellular localization. As expected, the GFP-tagged mature Aire protein was localized to the nucleus when expressed alone in MEFs (Fig. 5a). On the other hand, the mCherry-tagged immature Aire protein preferentially accumulated in the cytoplasm, owing to the lack of the nuclear localization signal (Fig. 5a). Interestingly, localization of mature Aire protein was changed from the nucleus to the cytoplasm when immature Aire protein was co-expressed (Fig. 5a). Exactly the same results were obtained when GFP- and mCherry-tags were exchanged (Supplementary Fig. 7). This finding led us to examine whether the presence of immature Aire protein affects stability of mature Aire protein. For this purpose, we developed HEK293 cells that constitutively express mature Aire protein, but inducibly express immature Aire protein when exposed to doxycycline (Fig. 5b). Doxycycline itself did not affect stability of mature Aire protein (Fig. 5c). However, the amount of mature Aire protein decreased, as the expression of immature Aire protein increased in response to doxycycline treatment (Fig. 5c). Since this reduction of mature Aire protein level was partially inhibited by treating cells with the proteasome inhibitor MG132 (Fig. 5d), it was suggested that mature Aire protein sequestrated into the cytoplasm becomes susceptible to proteasome-dependent protein degradation.

### A *cis*-regulatory element for intron retention of *Aire* gene

**Figure 6:**
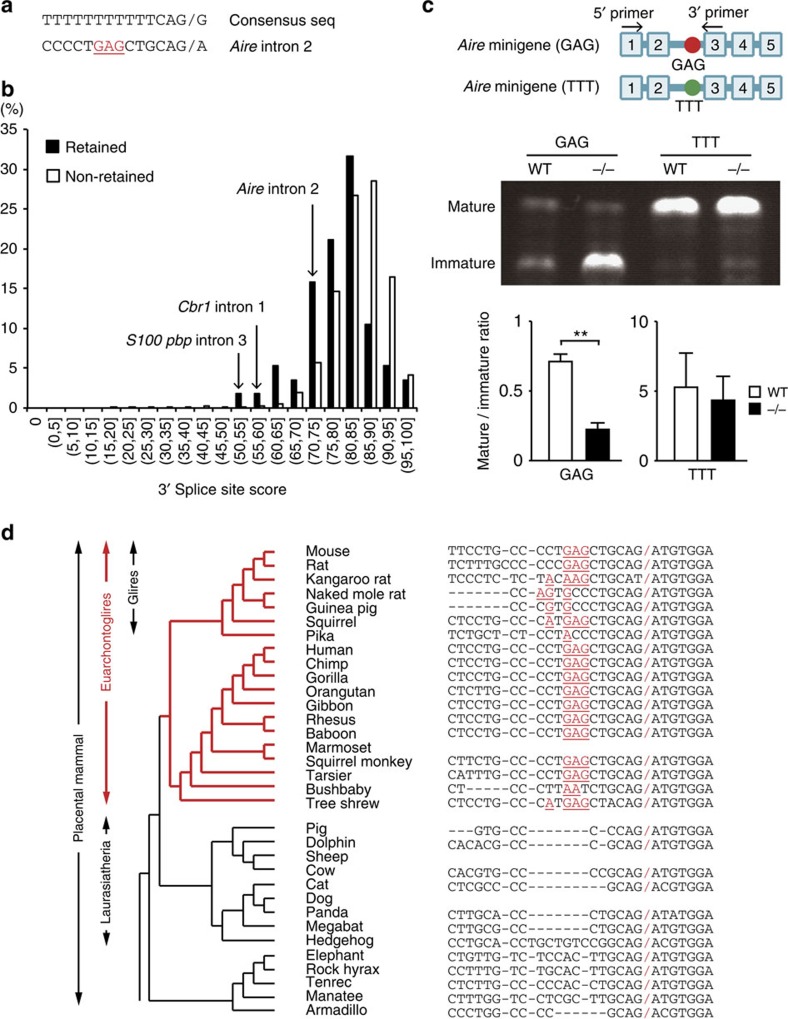
The 3′ splice site sequence is critical for intron 2 retention of *Aire* gene. (**a**) Comparison of 3′ splice site sequence of the *Aire* intron 2 with the consensus sequence. /, splice site. (**b**) Association between intron retention and 3′ splice site score revealed by comparing 57 retained introns with 188,151 unretained introns. (**c**) Effect of TTT mutation on intron 2 retention of minigene. Twenty-four hours after the transfection of WT and *Jmjd6*^*−/−*^ MEFs with GAG- or TTT-type minigene, the ratio of mature to immature transcript was analysed by RT-PCR (*n*=3). Data are collected from three separate experiments and are expressed as mean±s.d. **P<0.01 (two-tailed Student's *t*-test). (**d**) Comparison of the 3′ splice site sequence among different species. Non-canonical sequences at PPT site are indicated in red.

To understand why the intron 2 of *Aire* gene is susceptible to intron retention, we compared the sequence structure between Aire intron 2 and other introns. Although the most introns have the PPT site immediately upstream of the 3′ terminal AG dinucleotide^16^, *Aire* intron 2 encodes GAG instead of canonical pyrimidine-rich sequence at this position (Fig. 6a), resulting in ‘low' 3′ splice site score, which is calculated based on the similarity to the consensus sequence (Fig. 6a). Comparison of 57 retained introns with 188,151 unretained ones suggested that the degree of intron retention is associated with 3′ splice site score (Fig. 6b). Indeed, PCR with reverse transcription (RT-PCR) analyses revealed that intron 3 of *S100pbp* gene and intron 1 of *Cbr1* gene with low 3′ splice site score are preferentially retained in the absence of Jmjd6 (Supplementary Fig. 8).

To directly examine the effect of GAG sequence on the intron 2 retention, we created *Aire* minigene containing exons 1–5 surrounded by their intronic regulatory sequences with GAG or TTT at PPT site of the intron 2 (designated GAG-type or TTT-type) (Fig. 6c). When the GAG-type minigene was expressed in WT or *Jmjd6*^*−/−*^ MEFs, Jmjd6 deficiency markedly increased the transcript containing intron 2 (Fig. 6c), which was consistent with the results on endogenous *Aire* gene expression in mTECs and ES cells (Figs 3g and 4a). However, TTT-type minigene yielded only mature transcript without intron 2 retention, irrespective of Jmjd6 expression (Fig. 6c). Thus, GAG sequence acts as a *cis*-regulatory element that causes intron 2 retention and inhibits Aire protein expression. Interestingly, this GAG sequence at PPT site of *Aire* intron 2 is highly conserved in mammals in the Euarchontoglires clade (Fig. 6d). Therefore, intron retention may have been evolved as a mechanism to prevent overexpression of Aire protein in these species.

## Discussion

Intron retention is widely accepted as a consequence of mis-splicing, and its significance has been overlooked. However, recent evidence indicates that intron retention has a physiological role in some biological settings such as granulopoiesis^40^. Here we have demonstrated that the expression of Aire is controlled by Jmjd6 through intron retention. Although Jmjd6 deficiency did not affect abundance of *Aire* transcript, the intron 2 of *Aire* gene was not effectively spliced out in the absence of Jmjd6, resulting in a marked reduction of mature Aire protein in mTECs. In both *Jmjd6*^*−/−*^ E18.5 thymi and RANKL-stimulated *Jmjd6*^*−/−*^ fetal thymic stroma, the reduction of Aire protein was more prominent than that of mature *Aire* transcript. The exact reason for this discrepancy remains unclear. However, reconstitution experiments revealed that immature Aire protein generated by intron 2 retention affects subcellular localization and stability of mature Aire protein. Therefore, the ratio of mature Aire protein to the immature form might be a critical factor that determines expression and function of Aire protein in the nucleus.

Ectopic expression of peripheral TSAs by mTECs has been viewed as an essential mechanism for induction of central tolerance^3^,^4^,^5^,^6^,^7^,^8^. Consistent with a reduction of mature Air protein in mTECs, RNA-seq analyses revealed that the expressions of 23 Aire-dependent TSAs were markedly reduced in *Jmjd6*^*−/−*^ thymic stroma. This was further confirmed by quantitative real-time PCR analyses. In addition, by grafting *Jmjd6*^*−/−*^ thymic stroma into athymic C57BL/6 nude mice, we have shown that T-cells selected to mature in *Jmjd6*^*−/−*^ thymic microenvironments caused multi-organ autoimmunity. As the number of thymocytes was significantly reduced in the grafted *Jmjd6*^*−/−*^ thymus, disease manifestation in nu/nu^*Jmjd6−/−*^ mice might be exaggerated by homeostatic T-cell proliferation^41^. However, it has been reported that, while lymphocytes from Aire-deficient mice cause autoimmune disease when transferred into recombinase-activating gene (Rag)-deficient recipients, adoptive transfer of WT lymphocytes fail to induce disease under the same condition^5^,^15^. Thus, it is likely that reduction of Aire-dependent TSAs underlies disease development in nu/nu^*Jmjd6−/−*^ mice.

Mechanistically, our results suggest that Aire expression is tightly controlled via two discrete steps. First, owing to the GAG sequence at PPT site, the intron 2 of *Aire* gene is highly susceptible to intron retention in Euarchontoglires, and as a result, Aire protein expression is expected to be kept at a low level. As Aire has been reported to act as a proapoptotic factor^42^, this static regulation may be important to avoid a deleterious effect of Aire overexpression on the immune system or reproductive organs. The second important regulation is relief of intron retention. This is a dynamic process involving the enzymatic activity of Jmjd6, thereby raising the possibility that metabolic status and oxygen tension may influence intron retention. Although the direct substrate of Jmjd6 in this context is currently unknown, Jmjd6 interacts with multiple splicing regulatory proteins including U2 small nuclear ribonucleoprotein auxiliary factor 65 kDa (U2AF65)^22^,^23^. Therefore, it seems likely that Jmjd6 could alter affinity of a given splicing factor to 3′ splice site of *Aire* gene through lysyl hydroxylation. Our findings thus define a previously unknown mechanism controlling expression of Aire protein critical for establishment of immunological self in the thymus.

## Methods

### Mice

*Jmjd6*^*−/−*^ mice have been described previously^27^. Mice heterozygous for the mutant allele (*Jmjd6*^*+/–*^) were backcrossed onto a C57BL/6 background for more than 10 generations, and *Jmjd6*^*+/–*^ mice were crossed to obtain *Jmjd6*^*−/−*^ embryos. The morning of finding the vaginal plug was designated as E 0.5. B6.Cg-Foxn1<nu>/Nrs (nude) female mice were purchased from Taconic or provided by RIKEN BRC through National Bio-Resource Project of the MEXT, Japan and were used as recipients of thymic grafts at the age of 6–8 weeks. Mice were kept under specific pathogen-free conditions in the animal facility of Kyushu University. The protocol of animal experiments was approved by the committee of Ethics of Animal Experiments, Kyushu University.

### Fetal liver chimera

Fetal livers were harvested from E14.5 embryos. A total of 1 × 10^6^ fetal liver cells were injected in a volume of 300 μl phosphate-buffered saline (PBS) into the lateral tail veins of irradiated (10 Gy) CD45.1 C57BL/6 recipients. Chimeras were analysed 10 weeks after reconstitution.

### Fetal thymus organ culture (FTOC) and thymic graft

Thymic lobes were isolated from E15.5 WT and *Jmjd6*^*−/−*^ embryos, and were cultured for 4 days on Nucleopore filters (Whatman) placed in RPMI 1640 medium (Life Technologies) supplemented with 10% heat-inactivated fetal calf serum (FCS) (Nichirei Bioscience), 50 μM 2-mercaptoethanol (Nacalai tesque), 2 mM L-glutamine (Life Technologies), 100 U ml^–1^ penicillin (Life Technologies), 100 μg ml^–1^ streptomycin (Life Technologies), 1 mM sodium pyruvate (Life Technologies), MEM non-essential amino acids (Life Technologies) and 1.35 mM 2-DG (Sigma-Aldrich). After cultivation in complete RPMI medium without 2-DG for one more day, four pieces of WT and *Jmjd6*^*−/−*^ fetal thymi were grafted under the renal capsule of athymic (nude) and WT C57BL/6 mice. Thymic chimeras were analysed 6–10 weeks after transplantation. In some experiments, TECs were stimulated *in vitro* with recombinant RANKL (1 μg ml^–1^; Peprotech), agonistic anti-LtβR antibody 3C8 (2 μg ml^–1^; eBioscience) and/or recombinant CD40L (5 μg ml^–1^; R&D systems) for 4 days before analyses.

### Flow cytometry

The following antibodies and reagents were used at the indicated concentrations. Fluorescein isothiocyanate (FITC)-conjugated anti-mouse CD3ɛ (145-2c11, 5 μg ml^–1^), biotinylated anti-mouse CD4 (RM4–5, 5 μg ml^–1^), phycoerythrin (PE)-conjugated anti-mouse CD45 (30-F11, 1 μg ml^–1^), FITC-conjugated anti-mouse CD62L (MEL-14, 10 μg ml^–1^), FITC-conjugated anti-mouse CD90.2 (30-H12, 2.5 μg ml^–1^), FITC-conjugated anti-mouse Foxp3 (FJK-16 s, 5 μg ml^–1^), PE-conjugated anti-mouse MHC class II (I-A/I-E) (M5/114.15.2, 0.2 μg ml^–1^) and PerCP-Cyanine5.5 conjugated streptavidin (0.4 μg ml^–1^) were purchased from eBioscience. PE-conjugated anti-mouse CD8a (53–6. 7, 2 μg ml^–1^), PE-conjugated anti-mouse CD25 (PC61, 2 μg ml^–1^), PE-conjugated anti-mouse CD44 (IM7, 0.6 μg ml^–1^), FITC-conjugated anti-mouse CD45 (30-F11, 10 μg ml^–1^), FITC-conjugated CD45R/B220 (RA3-6B2, 10 μg ml^–1^), PE-conjugated anti-mouse CD80 (16–10A1, 1 μg ml^–1^), biotinylated anti-mouse CD45.2 (104, 5 μg ml^–1^) and APC-conjugated streptavidin (0.4 μg ml^–1^) were from BD Bioscience. Before staining with the antibodies, the cells were incubated for 10 min on ice with anti-Fcγ III/II receptor (2.4G2, 0.5 μg ml^–1^; BD Bioscience) to block Fc receptors. Foxp3 intracellular staining was performed with a staining kit (eBioscience) according to the manufacturer's recommendations. For intracellular Aire staining of mTECs, cells were first stained with anti-CD45 antibody and UEA-1 (10 μg ml^–1^; VECTOR Laboratories), followed by fixation in 4% (w/v) paraformaldehyde for 15 min at room temperature. After being permeabilized with 0.1% saponin (Sigma-Aldrich), cells were stained with Alexa Fluor 488-conjugated anti-mouse Aire (5H12, 5 μg ml^*–*1^; eBioscience) for 30 min on ice. Flow cytometric analyses were done on FACS Calibur (BD Bioscience).

### Histology and immunofluorescence

Tissues were fixed in 4% (w/v) paraformaldehyde and embedded in paraffin blocks. Sections (3 μm thick) were stained with haematoxylin and eosin, and examined by light microscopy. For immunofluorescence analyses, tissues were embedded in OCT compound (Sakura Finetech) and frozen at –80 °C. Cryostat sections (8 μm thick) were fixed by immersion for 10 min in ice-cold acetone and blocked with 10% horse serum (Sigma-Aldrich) for 1 h at room temperature. Sections were then incubated with rabbit anti-mouse keratin 5 (1 μg ml^*–*1^; Covance), rat anti-mouse keratin 8 (TROMA-1, 1:1,000 dilution; Developmental Studies Hybridoma Bank), biotinylated UEA-1 (10 μg ml^*–*1^; VECTOR Laboratories), Alexa Fluor 488-conjugated anti-mouse Aire (5H12, 2.5 μg ml^*–*1^; eBioscience) and/or PE-conjugated anti-mouse CD80 (16-10A1, 1 μg ml^*–*1^; eBioscience) for 1 h at room temperature. After being washed with PBS, sections were incubated with appropriately labelled secondary antibodies or reagents. For Jmjd6 staining, cells were cultured on the poly-L-lysine coated glass-bottom culture dishes (Matsunami), fixed with 4% (w/v) paraformaldehyde for 15 min, and permeabilized with 0.1% Triton X-100 for 1 h. After being blocked with 10% horse serum for 30 min at room temperature, cells were then stained with 4′,6-diamidino-2-phenylindole (DAPI) (Dojindo Laboratories), Alexa Fluor 546-conjugated phalloidin (Life Technologies), and mouse anti-Jmjd6 antibody (mAb328, 5 μg ml^*–*1^; Synaptic Systems) for 1 h at room temperature, followed by incubation with Alexa Fluor 488-conjugated donkey anti-mouse IgG (Fab fragment, 1.5 μg ml^*–*1^; Jackson ImmunoReseach). To detect autoantibodies, acetone-fixed cryosections of C57BL/6 nude mouse tissues were incubated with diluted serum (1:40) obtained from grafted mice, followed by staining with Alexa Fluor 488-conjugated anti-mouse IgG (4 μg ml^*–*1^; Life Technologies). All images were obtained with a laser scanning confocal microscope (LSM510 META; Carl Zeiss).

### Cell preparation and culture

To enrich TECs, thymic lobes were prepared from E18.5 or 6–8-week-old C57BL/6 mice, cut into small pieces, and dispersed further with pipetting to remove the majority of thymocytes. The resulting thymic fragments were digested with 0.125% (w/v) collagenase D/dispase (Roche) and 0.1% (w/v) DNase I (Roche) in RPMI1640 medium for 1 h at 37 °C. The supernatants containing dissociated TECs were centrifuged and were washed with PBS. Before cell sorting, TECs were further enriched by depleting CD45^+^ haematopoietic cells using CD45 MicroBeads (Miltenyi Biotec), and stained with the relevant antibodies and reagents. Then, mTECs and cTECs were sorted as CD45^*–*^ MHC class II^*+*^ UEA-1^+^ cells and CD45^*–*^ MHC class II^*+*^ UEA-1^*–*^ cells, respectively. ES cells were developed from E3.5 blastocysts by using standard procedures. Integrity of ES cells was confirmed by staining them for alkaline phosphatase with a kit (Wako Pure Chemical Industries). ES cells were cultured in knockout D-MEM medium (Life Technologies) supplemented with 15% FCS (Life Technologies), 50 μM 2-mercaptoethanol (Nacalai tesque), 2 mM L-glutamine (Life Technologies), 100 U ml^–1^ penicillin (Life Technologies), 100 μg ml^–1^ streptomycin (Life Technologies) and ESGRO leukemia inhibitory factor (LIF) (Millipore) at final concentration of 1,500 units per ml. On the other hand, primary MEFs were generated from E13.5 WT and *Jmjd6*^−/−^ embryos. Primary MEFs were immortalized by transfection with a plasmid pCX4bsr-SV40ER (provided by T. Akagi, KAN Research Institute, Kobe, Japan). Immortalized MEFs were cultured in D-MEM medium (Wako Pure Chemical Industries) supplemented with 10% FCS (Nichirei Bioscience), 100 U ml^–1^ penicillin (Life Technologies) and 100 μg ml^–1^ streptomycin (Life Technologies).

### RNA-seq analysis and 3′ splice site scoring

Total RNA was extracted from WT and *Jmjd6*^−/−^ FTOC samples with (two samples for each category) or without (one sample for each category) RANKL stimulation. One mcirogram of total RNA was used for library construction with TruSeq RNA Sample Prep kit v2 (Illumina) according to the manufacture's protocol. Briefly, poly-A-containing mRNAs were purified using poly-T oligo-attached magnetic beads. The purified mRNAs were fragmented using divalent cations under elevated temperatures and then converted to dsDNA by two rounds of cDNA synthesis using reverse transcriptase and DNA polymerase I. After an end repair process, DNA fragments were ligated with adaptor oligos. The ligated products were amplified by eight cycles of PCR to generate RNA-seq library. Library integrity was verified by Bioanalyzer DNA1000 assay (Agilent Technologies). Sequencing was performed in 101-bp paired-end mode using an Illumina HiSeq (Illumina). A total of 177,060,020 reads were obtained for six samples. Filtered reads were mapped to the UCSC mm10 using the TopHat program (v2.0.10)^43^ with the default parameters. The Cufflinks program (v2.1.1)^44^ was then used to assemble 22,448 transcripts and to calculate the fragments per kilobase of exon per million mapped fragments (FPKM) values, which are normalized measurement of gene expression levels, with the non-default parameters: -u—library-type fr-secondstrand. To identify differentially expressed genes, the ratio of the maximum FPKM to the minimum FPKM was compared among six samples. When the ratio was more than 3, the gene was regarded as being significantly altered in expression level. We added 0.1 to the FPKM value to avoid division by 0. This led us to identify 3,212 genes with differential expression. Among these, the expression levels of 2,536 genes were significantly associated with either RANKL treatment or Jmjd6 expression (*P*-value<0.05), and these genes were used for further analyses. Analysis of intron retention was performed as follows. According to the current gene annotation (‘known genes' in UCSC mm10), there are 188,208 introns in total. As intron retention events should be observed in the genes with relatively high expression, we only focused on the genes with the maximum FPKM value more than 10 at least in one of the six samples. As a result, we obtained 84,708 introns. The reads mapped to these intronic regions were counted by the intersectBed program in the BEDTools utilities (v2.17.0)^45^ with –c option, and the counts are converted into the FPKM values for each intron (intronic FPKM). There are 1,051 introns with intronic FPKM more than 10 for at least one of the six samples, and the degree of intron retention (IR value) was calculated by dividing intronic FPKM value by conventional FPKM value for each gene. By filtering IR value of *Jmjd6*^−/−^ sample to that of WT sample more than 1.5, we finally selected 57 introns that are preferentially expressed in *Jmjd6*^−/−^ samples under RANKL stimulation. The 3′ splice site score was calculated by ‘Splice-Site analyser tool' (http://ibis.tau.ac.il/ssat/SpliceSiteFrame.htm)^46^. Shortly, the score expresses to what extent the splice-site sequences match the following consensus sequence: TTTTTTTTTTTCAG/G (‘/' indicate the intron/exon junction).

### RT-PCR and Southern blot analysis

After treatment with RNase-free DNase I (Life Technologies), RNA samples were reverse-transcribed with oligo(dT) primers (Life Technologies) and SuperScript III reverse transcriptase (Life Technologies) for amplification by PCR. The following PCR primers were used for RT-PCR and/or Southern blot analysis. *Aire* (exon 1 – exon 10); 5′-AGGTGGGGATGGAATGCTA-3′ and 5′-CCTCATTCCCAGCACTCAGTA-3′. *Aire* (exon 1 – exon 3); 5′-TAGACAGTGCCTTTCCGCTGCTGC-3′ and 5′-GGAGACGCTCTTTGAGGCCAGAGTTG-3′. *Actb*; 5′-TGGAATCCTGTGGCATCCATGAAAC-3′ and 5′-TAAAACGCAGCTCAGTAACAGTCCG-3′. *Jmjd6*; 5′-ACCACAAGAGCAAGAAGCGCATCC-3′ and 5′- AACCACGGGCTTGTAAGGCCTC-3′. *S100pbp*; 5′-TCCTCCAAAGAAACGGAAAA-3′, 5′-CCTAAGTGCGGGGATTACAA-3′, and 5′-TCCCCAGAATCCTTGTCAAA-3′. *Cbr1*; 5′- GGTGACCGGTGCTAACAAAG-3′, 5′-ATTGGGTGTGGAGATGGAAG-3′, and 5′-TCACCTCTGCTTGAATGTGG-3′. For Southern blot analysis, *Sac* I-*Sac* II fragment of *Aire* cDNA (560 bp-fragment corresponding to exons 3–8) was used as a probe. Quantification of the intensity was performed with BAS-2500 bio-imaging analyser and the Image Gauge 4.0 software (Fujifilm CO.). Identity of all detected bands was confirmed by sequencing.

Real-time PCR was performed on ABI PRISM 7,000 Sequence Detection System using the SYBR Green PCR Master Mix (both from Applied Biosystems). The following PCR primers were used: *Gapdh*; 5′-TGTGTCCGTCGTGGATCTGA-3′ and 5′-TTGCTGTTGAAGTCGCAGGAG-3′. *Aire*; 5′-GAAGCCAGATGGCAACTTGGA-3′ and 5′-ACAGAAGCTGCCATGGTCTGAA-3′. *Psmb11*; 5′-CTCTGTGGCTGGGACCACTC-3′ and 5′-TCCGCTCTCCCGAACGTGG-3′. *Col1a2*; 5′-CCGTGCTTCTCAGAACATCA-3′ and 5′-GAGCAGCCATCGACTAGGAC-3′. *Jmjd6*; 5′-AGAAACGCAGACCCCCTTAC-3′ and 5′-CCCTGAACTAAGGCATTCCA-3′. *Ins2*; 5′-CCACCAGCCCTAAGTGATCC-3′ and 5′-TAGAGAGCCTCCACCAGGTG-3′. *Spt1*; 5′-ACTCCTTGTGTTGCTTGGTGTTT-3′ and 5′-TCGACTGAATCAGAGGAATCAACT-3′. *Gad1*; 5′-CACCTGGAACCCTCACAA-3′ and 5′-ACTGCTTGTCTGGCTGGA-3′. All targets were standardized to *Gapdh* signals.

### Plasmids and transfection

For expression of N-terminally-tagged GFP- or mCherry- fusion proteins in mammalian cells, expression vector was created by subcloning a cDNA encoding EGFP or mCherry into pCI (Promega). The genes encoding the full-length Aire protein (mature Aire) and the truncated Aire protein (immature Aire) containing only exon 1 and exon 2 owing to intron 2 retention were subcloned into these vectors to analyse their subcellular localization in MEFs. Transfection was performed with Lipofectamine 2,000 reagent (Life Technologies). To create inducible expression system, the gene encoding HA-tagged immature Aire protein was subcloned in the pTRE2hyg vector (Clonetech) (designated pTRE2hyg-HA-immature Aire). After electroporation of pCI-GFP-mature Aire and pTRE2hyg-HA-immature Aire or pTRE2hyg into HEK293 Tet-On Advanced cells (Clonetech), cells were selected with 150 μg ml^–1^ hygromycin B (Wako Pure Chemical Industries) to develop stable transfectants. *Aire* minigene construct (GAG-type; 2.4 kb) was amplified using mouse genome DNA and subcloned into TAget Clone TM Plus (TOYOBO Co. Ltd.). After digestion with *Sal* I and *Not* I, the minigene construct was inserted into pSI vector (Promega) for transfection. The TTT-type minigene was generated by site-directed mutagenesis. The gene encoding the C-terminally GFP-tagged WT Jmjd6 was subcloned into pCI vector (designated pCI-Jmjd6-GFP). The 5 A mutant encoding alanines instead of H187, D189, K204, T285 and N287 was generated by site-directed mutagenesis. For splicing assay, *Aire* minigene plasmids (1 μg) was transfected into immortalized WT or *Jmjd6*^−/−^ MEFs (2 × 10^5^ cells) in 6-well plates with Lipofectamine 2000 reagent. To express Jmjd6 and its mutant in *Jmjd6*^−/−^ ES cells, these ES cells were electroporated with pCI-Jmjd6-GFP vector (WT or 5 A mutant) by using the Mouse ES Cell Nucleofector Kit (Lonza/Amaxa Biosystems). The GFP-positive ES cells were then sorted by FACSAria for RNA extraction 30 h after transfection.

### Immunoblotting

Cells were lysed on ice in 20 mM Tris-HCl buffer (pH 7.5) containing 1% Triton X-100, 150 mM NaC1, 1 mM EDTA, 1 mM EGTA, 2.5 mM sodium pyrophosphate, 1 mM β-glycerophosphate, 1 mM Na_3_VO_4_), and complete protease inhibitors (Roche). After centrifugation, the supernatants were mixed with an equal volume of 2 × sample buffer (125 mM Tris-HCl, 0.01% bromphenacyl bromide, 4% SDS, 20% glycerol and 200 μM dithiothreitol). Samples were boiled for 5 min and analysed by immunoblotting. The following antibodies were used: goat anti-actin (I-19; 1:1,000 dilution; Santa Cruz), mouse anti-GFP (B-2; 1:1,000 dilution; Santa Cruz), and rat anti-HA (3F10; 1:500 dilution; Roche). Before assays, HEK293 cells stably expressing both pCI-GFP-mature Aire and pTRE2hyg-HA-immature Aire or pTRE2hyg were treated with doxycycline (Dox; 10 μg ml^–1^; Clonetech) in the presence or absence of MG132 (10 μM; Sigma-Aldrich) for specified times. The signals were measured using ImageJ public-domain software (imagej.nih.gov/ij/) and standardized to β-actin. In Fig. 5c,d, images have been cropped for presentation. Full size images are presented in Supplementary Figure 9.

### Hydroxylation assays

Hydroxylation assays were performed as previously described^22^,^39^. Briefly, the assays consisted of the substrate mixture: LUC7-like2 peptide (LUC7L2_267–278_; 100 μM), 2-oxoglutarate (500 μM), ascorbate (100 μM) in Tris (50 mM, pH 7.5) and the enzyme mixture: FeNH_4_SO_4_ (100 μM) and recombinant Jmjd6 (100 μM) in Tris (50 mM, pH 7.5). The reaction was initiated by mixing the substrate and enzyme mixtures in a final volume of 20 μl and by incubating at 37 °C for 1 h. The reaction was quenched by adding 10 μl of 0.1% (w/v) CF_3_CO_2_H. Mass spectrometry analysis was performed using an Autoflex matrix-assisted laser desorption/ionization (MALDI) time-of-flight (TOF) mass spectrometer (Bruker Daltonics). The quenched reaction mixture was diluted 10-fold with 0.1% CF_3_CO_2_H and 2% acetonitrile, 1 μl of which was directly spotted onto 0.5 μl of α-cyano-4-hydroxycinnamic acid matrix on the MALDI target plate and allowed to dry. The mass corresponding to LUC7L2_267–278_ and LUC7L2_267–278_ +16, equivalent to one hydroxylation, were identified.

### Statistical analysis

For statistical analysis, *P* values were calculated with a two-tailed unpaired Student's *t*-test. *P* values <0.05 were considered significant. Error bars denote±s.d.

## Additional information

**Accession codes:** RNA-seq data have been deposited in the gene expression omnibus (GEO) database under accession code GSE61444.

**How to cite this article:** Yanagihara, T. *et al*. Intronic regulation of Aire expression by Jmjd6 for self-tolerance induction in the thymus. *Nat. Commun.* 6:8820 doi: 10.1038/ncomms9820 (2015).

## Supplementary Material

Supplementary Information Supplementary Figures 1-9 and Supplementary Tables 1-2
