# Supplementary material for: Intronic regulation of Aire expression by Jmjd6 for self-tolerance induction in the thymus
Source: Nat Commun. 2015 Nov 4;6:8820. doi: 10.1038/ncomms9820 (PMC4667615; doi:10.1038/ncomms9820)
Supplement: Supplementary Information — Supplementary Figures 1-9 and Supplementary Tables 1-2 [file ncomms9820-s1.pdf]

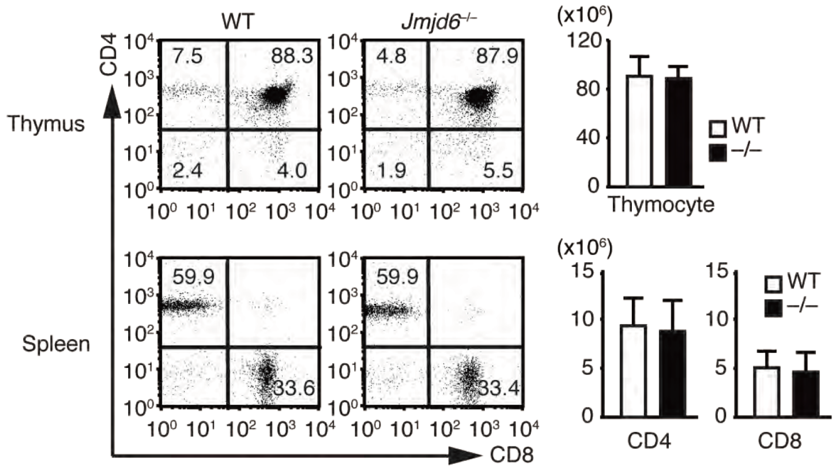

Supplementary Figure 1 │ Normal development of *Jmjd6*$^{-/-}$ thymocytes in chimeric mice reconstituted with fetal liver cells from the knockout mice. 10 weeks after reconstitution of CD45.1 recipients with CD45.2 fetal liver cells from WT and *Jmjd6*$^{-/-}$ littermates (n = 5), thymocytes and spleen cells were stained with antibodies specific for CD45.2, CD90.2, CD4 and/or CD8. The numbers of CD45.2^{+} thymocytes, CD4^{+} T cells and CD8^{+} T cells were analysed.

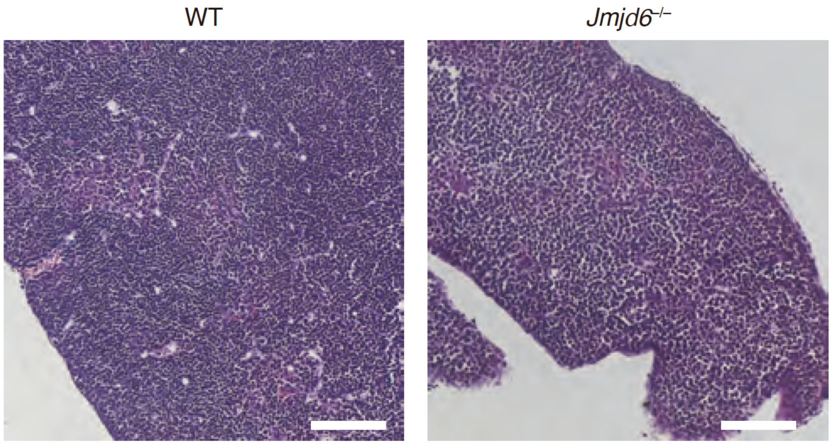

Supplementary Figure 2 | Small, but normal architecture of the thymus from *Jmjd6*$^{-/-}$ embryos. Haematoxylin and eosin staining of the thymus sections from E18.5 WT (*Jmjd6*$^{+/+}$) and *Jmjd6*$^{-/-}$ embryos. Scale bars, 100 μm. Data are representative of three independent experiments.

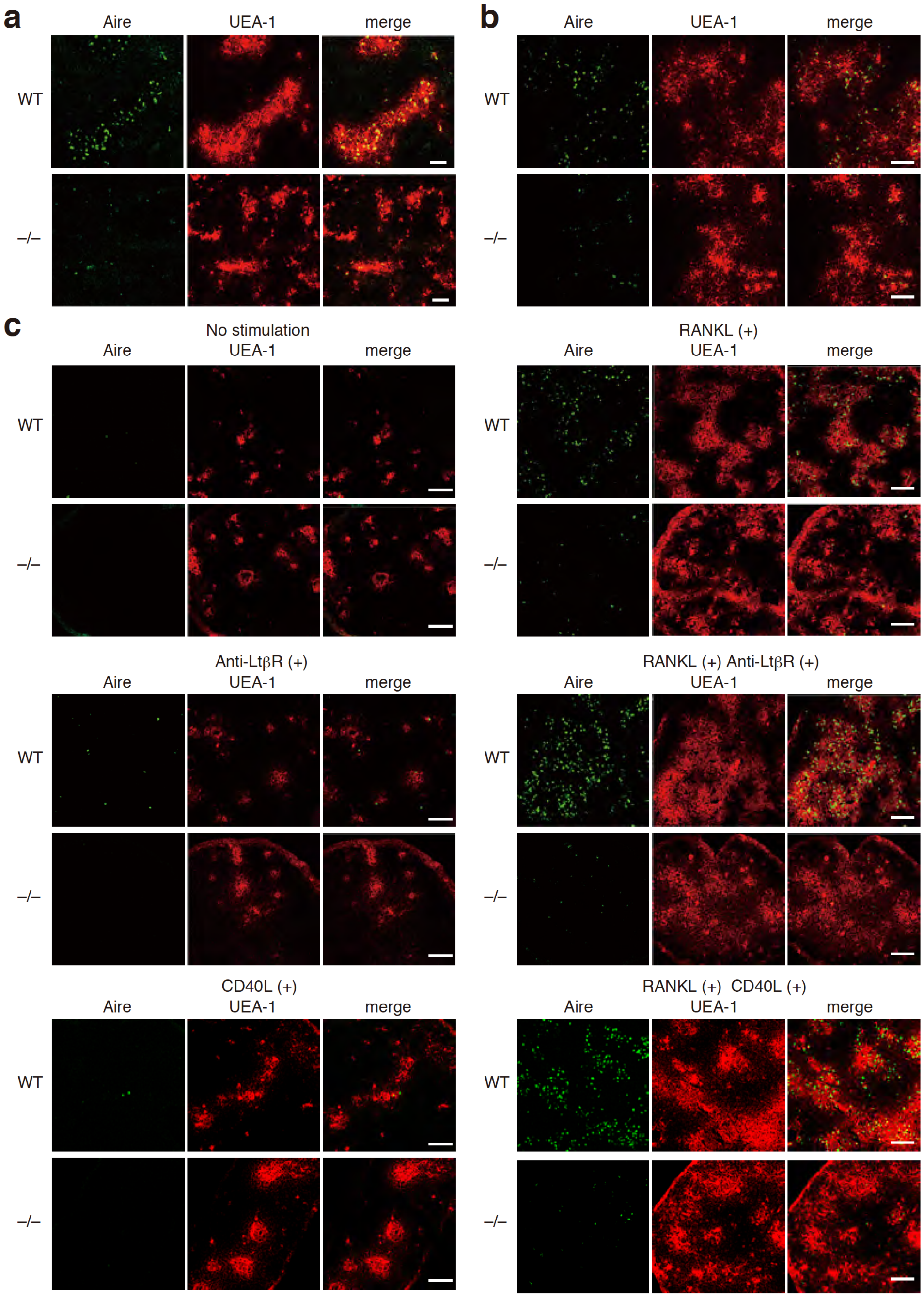

Supplementary Figure 3 | Single colour images for Aire protein expression in mTECs. E18.5 thymi (**a**), thymi grafted under the kidney capsule of C57BL/6 mice (**b**), and 2-DG-treated fetal thymic stroma stimulated with RANKL, anti-LtβR antibody and/or CD40L were stained with UEA-1 and anti-Aire antibody. Scale bars, 50 μm.

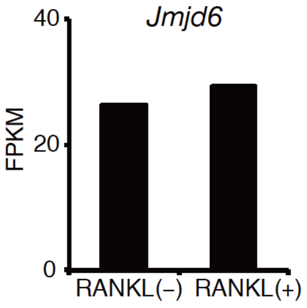

Supplementary Figure 4 │ Comparable expression of *Jmjd6* in fetal tymic stroma between before and after RANKL stimulation. Data were obtained from RNAseq analyses for WT samples.

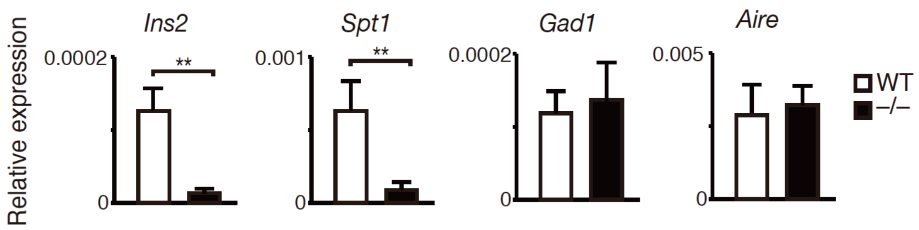

Supplementary Figure 5 │ Comparison of gene expression of Aire-dependent and -independent TSAs in E18.5 thymi. The expressions of *Ins2*, *Spt1*, *Gad1*, and *Aire* were compared between WT ($n = 9$) and *Jmjd6*$^{-/-}$ ($n = 4$) thymi at E18.5 by real-time PCR. Data are expressed as mean ± s.d. $^{**}P < 0.01$ (two-tailed Student's t-test).

Exon 1 | Exon 2 | Intron 2 | Exon 3

```
ATGGCAGGTGGGGATGGAATGCTACGCCGTCTGCTGAGGCTGCACCGCACCGAGATCGCG
 M  A  G  G  D  G  M  L  R  R  L  L  R  L  H  R  T  E  I  A

GTGGCCATAGACAGTGCCTTTCCGCTGCTGCATGCTCTAGCCGACCACGACGTGGTCCCT
 V  A  I  D  S  A  F  P  L  L  H  A  L  A  D  H  D  V  V  P

GAGGACAAGTTCCAGGAGACGCTCCGTCTGAAGGAGAAGGAAGGCTGCCCCCAGGCCTTC
 E  D  K  F  Q  E  T  L  R  L  K  E  K  E  G  C  P  Q  A  F

CACGCCCTGCTGTCCTGGCTCCTGACCCGGGACAGTGGGGCCATCCTGGATTTCTGGAGG
 H  A  L  L  S  W  L  L  T  R  D  S  G  A  I  L  D  F  W  R

ATTCTCTTTAAGGACTACAATCTGGAGCGGTACAGCCGCCTGCATAGCATCCTGGACGGC
 I  L  F  K  D  Y  N  L  E  R  Y  S  R  L  H  S  I  L  D  G

TTCCCAAAAGGTGGGCGTGTGCTGATTGATGCTGGAGCTGATGCTCAGCCAATGGGTAGC
 F  P  K  G  G  R  V  L  I  D  A  G  A  D  A  Q  P  M  G  S

ATCGGGGATATGGATACAAGTCGGCCCATGTTTTCAGGGAGCCACTAGAACTTGGGCAGA
 I  G  D  M  D  T  S  R  P  M  F  S  G  S  H  *  N  L  G  R

TCCTAAGAAGCAAAGGGCAGAGGTCTGCTCTTTCTCGTCCTCAAGAGTGCCCCATTCTAG
 S  *  E  A  K  G  R  G  L  L  F  L  V  L  K  S  A  P  F  *

AGCTCACCCTGAAGATAAGGCTTTAAGACAGGACCATTGTTCCTGCCCCTGAGCTGCAGA
 S  S  P  *  R  *  G  F  K  T  G  P  L  F  L  P  L  S  C  R

TGTGGACCTAAACCAGTCCCGGAAAGGGAGAAAGCCCCTTGCTGGTCCCAAGGCCGCGGT
 C  G  P  K  P  V  P  E  R  E  K  A  P  C  W  S  Q  G  R  G
```

Supplementary Figure 6 | The intron 2 retention of *Aire* gene results in an appearance of a premature termination codon. Predicted amino acid sequence of immature Aire protein generated by retention of intron 2 is shown.

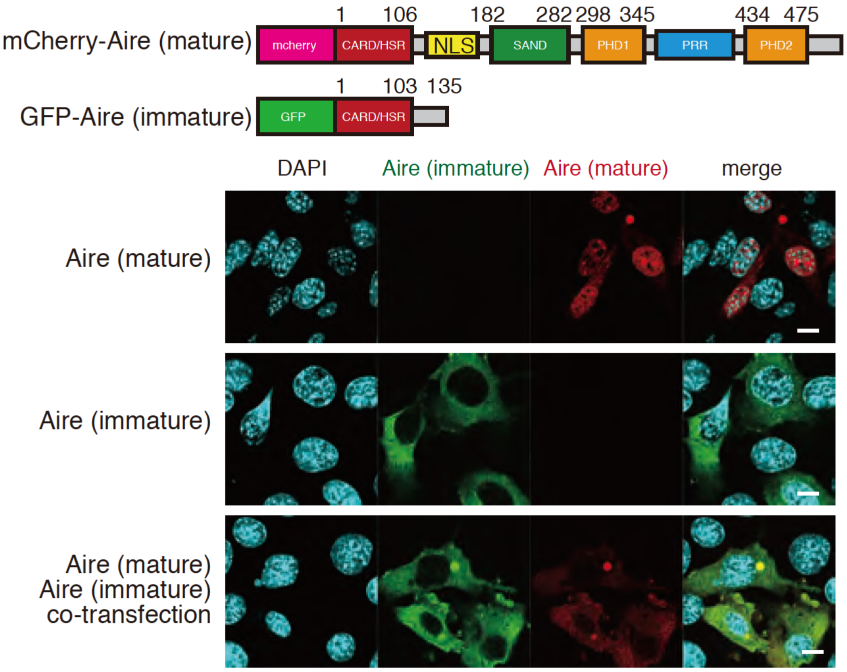

Supplementary Figure 7 | Subcellular localisation of mature and immature Aire proteins. Following expression of mCherry-tagged mature Aire protein and GFP-tagged immature Aire protein in MEFs, their localisation was analysed by confocal microscopy. Scale bars, 10 μm. CARD: caspase recruitment domain; HSR: homogenously staining region; SAND: Sp100, Aire, NucP41/75, DEAF1 domain; PHD: plant homeodomain; PRR: proline-rich region.

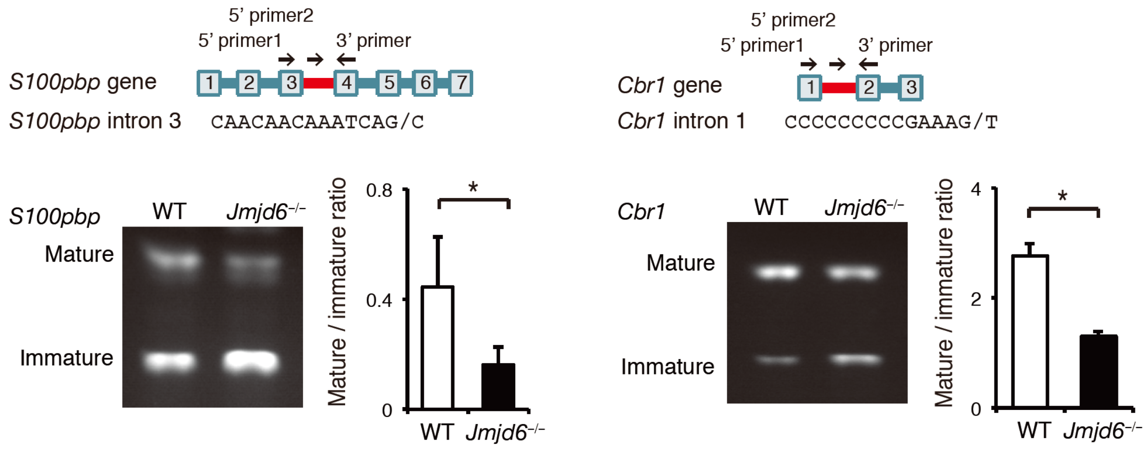

Supplementary Figure 8 | Intron retention of other genes with low 3’ splice site score. The degrees of intron retention of *S100pbp* gene (intron 3) and *Cbr1* gene (intron 1) were compared between RANKL-stimulated WT (n = 4 for *S100pbp* gene and n = 3 for *Cbr1* gene) and *Jmjd6*$^{-/-}$ (n = 4 for *S100pbp* gene and n = 3 for *Cbr1* gene) thymic stroma. Data are expressed as mean ± s.d. $*P < 0.05$ (two-tailed Student’s *t*-test).

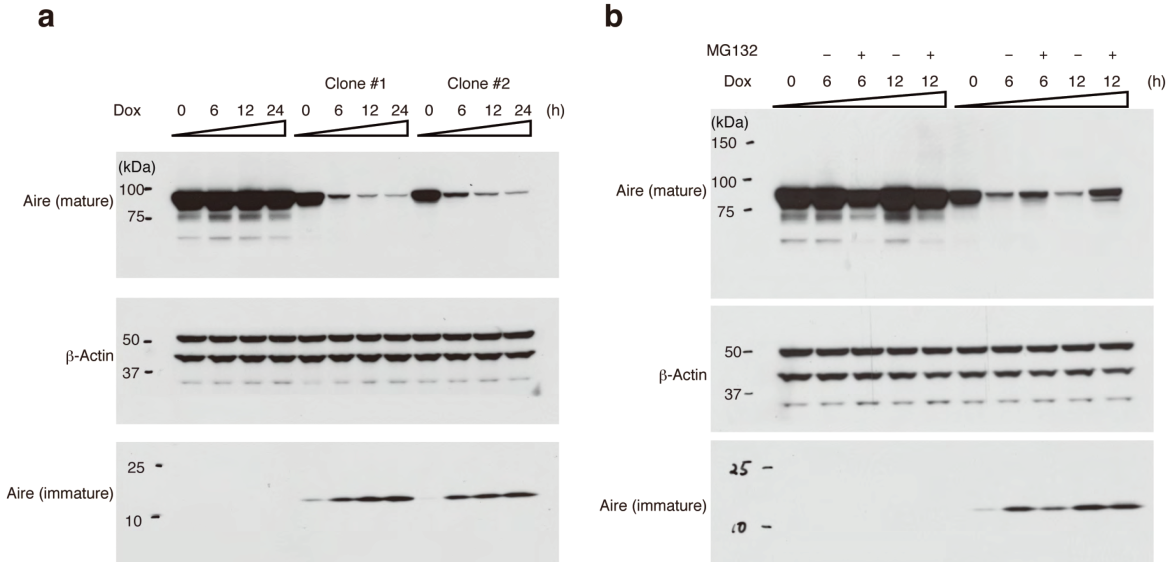

Supplementary Figure 9 │ Full size images for Western blots used in Figure 5.

Supplementary Table 1 │ Top 200 genes that are induced in WT thymic stroma in response to RANKL stimulation at higher levels than those in *Jmjd6*$^{-/-}$ stroma

List number: 1 ~ 100

List number	Gene name	Samples with RANKL stimulation			List number	Gene name	Samples with RANKL stimulation		
		The ratio of Jmjd6$^{-/-}$ FPKM value to that of WT sample	FPKM value for WT sample	FPKM value for Jmjd6$^{-/-}$ sample			The ratio of Jmjd6$^{-/-}$ FPKM value to that of WT sample	FPKM value for WT sample	FPKM value for Jmjd6$^{-/-}$ sample
1	*Slc17a4*	0	0.2393395	0	51	*Gm5771*	0	0.310677	0
2	*Gm10696*	0	0.194387	0	52	*Ear11*	0	0.1862115	0
3	*H2-M10.1*	0	0.220702	0	53	*Sohlh1*	0	0.4038855	0
4	*Ifnl2*	0	0.493682	0	54	*Fam151a*	0	0.217763	0
5	*Oas1d*	0	0.687804	0	55	*Lcn8*	0	1.1112625	0
6	*Kif12*	0	0.3690585	0	56	*Defa21*	0	0.5229125	0
7	*Apoc4*	0	0.3601495	0	57	*Insulin 2*	0	0.537473	0
8	*AY761184*	0	0.225383	0	58	*Defb23*	0	0.2143975	0
9	*Pklr*	0	0.3525905	0	59	*MARCH11*	0	0.14406485	0
10	*Cabp2*	0	0.4008165	0	60	*Tff2*	0	1.85747	0
11	*Amtn*	0	0.742071	0	61	*Notum*	0	0.2639455	0
12	*Klk15*	0	0.28998	0	62	*Gm19299*	0	0.14617355	0
13	*Reg4*	0	0.462469	0	63	*Pnmt*	0	0.812358	0
14	*Lrg1*	0	0.1863345	0	64	*1700020A23Rik*	0	0.231737	0
15	*1700011B04Rik*	0	0.2780575	0	65	*C030013G03Rik*	0	0.14673445	0
16	*Crisp3*	0	0.452326	0	66	*Sval1*	0	0.59171	0
17	*Noxa1*	0	0.221082	0	67	*1810065E05Rik*	0	0.7519035	0
18	*Fam162b*	0	0.6307835	0	68	*Klk1b3*	0	0.405906	0
19	*Cst10*	0	0.2183705	0	69	*Mcpt-ps1*	0	0.242476	0
20	*Wfdc1*	0	0.3242705	0	70	*Tomm20l*	0	0.21653595	0
21	*Mbl2*	0	0.482808	0	71	*Ceacam9*	0	0.2947765	0
22	*Defb19*	0	0.2458765	0	72	*Pla2g5*	0	0.16983225	0
23	*Dcpp2*	0	0.5443815	0	73	*Ctrb1*	0	0.14435305	0
24	*Cst8*	0	0.432606	0	74	*Phgr1*	0	0.4815455	0
25	*Rgs11*	0	0.504684	0	75	*Slc51b*	0	0.234606	0
26	*Ido1*	0	0.2296315	0	76	*Akr1c21*	0	0.1996526	0
27	*Wap*	0	0.294191	0	77	*Saa2*	0	2.0114305	0
28	*Il9*	0	0.6883585	0	78	*Erp27*	0	0.2617905	0
29	*Prap1*	0	2.05573	0	79	*Rbp7*	0	0.4807305	0
30	*Spink4*	0	1.155066	0	80	*Mcpt2*	0.014932256	4.355705	0.0650405
31	*Khdc3*	0	0.3752395	0	81	*Mcpt4*	0.015397172	1.3603245	0.02094515
32	*Bpifb9a*	0	0.1881405	0	82	*Retn*	0.018173169	3.347545	0.0608355
33	*Fgf8*	0	0.2389905	0	83	*Mcpt1*	0.01839071	6.466235	0.11891865
34	*Sstr5*	0	0.1603945	0	84	*Csn1s2a*	0.018848975	1.21483	0.0228983
35	*Tspan32*	0	0.2340405	0	85	*Mgarp*	0.019981692	3.042445	0.0607932
36	*Reg3a*	0	1.1383735	0	86	*Mybpc3*	0.02070688	0.2417385	0.00500565
37	*Agxt*	0	0.154671	0	87	*Reg3b*	0.022743404	8.1911	0.1862935
38	*Prm1*	0	0.426924	0	88	*Itih3*	0.023649018	0.2719965	0.00643245
39	*Tmem171*	0	0.1559485	0	89	*Miat*	0.02578377	0.32847	0.008469195
40	*Ldlrad1*	0	0.1938095	0	90	*Prss34*	0.02603249	0.8089545	0.0210591
41	*Psg22*	0	0.207911	0	91	*Prss29*	0.026166518	0.8544985	0.02235925
42	*Bglap*	0	0.3642855	0	92	*Myh8*	0.026823214	0.1474141	0.00395412
43	*Cma1*	0	0.711497	0	93	*Dcpp3*	0.029120609	1.475965	0.042981
44	*Tpsg1*	0	0.926741	0	94	*Gsdma2*	0.02981626	0.3990155	0.01189715
45	*Rpl39l*	0	0.5351985	0	95	*Nlrp6*	0.031811884	0.1981115	0.0063023
46	*1810008I18Rik*	0	0.215145	0	96	*Bpifb3*	0.032955823	0.4387965	0.0144609
47	*Gm1332*	0	0.2247265	0	97	*Gzmb*	0.033407003	0.5585775	0.0186604
48	*H2-T3*	0	0.15118875	0	98	*Clec4f*	0.033929663	0.3362235	0.01140795
49	*Nhlrc4*	0	0.2379935	0	99	*Lrcol1*	0.034258865	1.025771	0.03514175
50	*Pla2g1b*	0	0.675617	0	100	*Reg1*	0.034551211	4.78688	0.1653925

List number: 101 ~ 200

List number	Gene name	Samples with RANKL stimulation			List number	Gene name	Samples with RANKL stimulation		
		The ratio of Jmjd6$^{-/-}$ FPKM value to that of WT sample	FPKM value for WT sample	FPKM value for Jmjd6$^{-/-}$ sample			The ratio of Jmjd6$^{-/-}$ FPKM value to that of WT sample	FPKM value for WT sample	FPKM value for Jmjd6$^{-/-}$ sample
101	*Cryaa*	0.035634012	1.327775	0.04731395	151	*Lefty2*	0.068386496	1.275485	0.08722595
102	*Ibsp*	0.036173989	0.4905265	0.0177443	152	*Sct*	0.070779939	1.984065	0.140432
103	*Bhlhe23*	0.037457902	0.4315805	0.0161661	153	*T*	0.070918917	0.198734	0.014094
104	*Spt1*	0.037834757	6.35199	0.240326	154	*Spaca1*	0.071036621	0.2964295	0.02105735
105	*Dcpp1*	0.037995405	2.870755	0.1090755	155	*Igfbp6*	0.0714433	2.10476	0.150371
106	*Klk1*	0.040209164	1.7546	0.070551	156	*Crh*	0.071577697	0.44828	0.03208685
107	*Rln3*	0.040422778	2.085965	0.0843205	157	*P2rx1*	0.073116299	0.409566	0.02994595
108	*Psg21*	0.042611511	0.216323	0.00921785	158	*Sst*	0.073373987	4.858125	0.35646
109	*Pgc*	0.042653356	2.88561	0.12308095	159	*Psg27*	0.0734766	0.7855535	0.0577198
110	*Apoc3*	0.043391882	1.37891	0.0598335	160	*Mrgprg*	0.075686901	0.2534025	0.01917925
111	*Gsdmc3*	0.043405126	0.872511	0.03787145	161	*Actc1*	0.075976828	0.291812	0.02217095
112	*Birc7*	0.044135965	0.458737	0.0202468	162	*Serpina3c*	0.0763775	0.1810435	0.01382765
113	*Ccl1*	0.045423607	1.358985	0.06173	163	*Hand1*	0.076754213	0.768849	0.0590124
114	*Slc51a*	0.045774394	0.8928365	0.04086905	164	*Ceacam18*	0.076815453	0.8907075	0.0684201
115	*Lrrc4b*	0.046196808	0.258546	0.011944	165	*Oas1e*	0.077375455	0.8298885	0.064213
116	*Amelx*	0.046993527	0.7368685	0.03462805	166	*Itih4*	0.077817247	0.326977	0.02544445
117	*Car4*	0.047183587	1.197942	0.0565232	167	*Klhl40*	0.078571956	0.4468	0.03510595
118	*Hcrtr1*	0.047354475	0.2847355	0.0134835	168	*Dppa3*	0.079501107	2.27644	0.1809795
119	*Myl3*	0.048357018	0.6238505	0.03016755	169	*1700029F12Rik*	0.079760803	0.2339075	0.01865665
120	*Cym*	0.04900222	0.384754	0.0188538	170	*Fabp1*	0.079967447	1.237985	0.0989985
121	*Crabp1*	0.049574777	5.154235	0.25552005	171	*Ucma*	0.080126175	5.26172	0.4216015
122	*Cst9*	0.050363118	0.7199455	0.0362587	172	*Ppp1r17*	0.082110843	0.1349499	0.01108085
123	*Oas1h*	0.050626177	1.202535	0.06087975	173	*Tmem92*	0.082330899	0.455275	0.0374832
124	*Prss1*	0.050824751	0.671112	0.0341091	174	*Gm8267*	0.082735597	0.178451	0.01476425
125	*Pck1*	0.051877981	0.378039	0.0196119	175	*Retnla*	0.08287184	1.64466	0.136296
126	*Skint11*	0.052656304	0.250329	0.0131814	176	*Obp2a*	0.083688752	0.384382	0.03216845
127	*Gsdmc2*	0.054613518	1.39502	0.07618695	177	*Pdyn*	0.083714585	0.483583	0.04048295
128	*Cyp1a2*	0.054925875	0.2970995	0.01631845	178	*Alms1-ps2*	0.083943094	0.4558755	0.0382676
129	*2210407C18Rik*	0.056771328	4.26615	0.242195	179	*Crtac1*	0.085577319	0.589509	0.0504486
130	*Alpi*	0.056957943	0.3403125	0.0193835	180	*Mpo*	0.088291597	1.301215	0.11488635
131	*Sec14l3*	0.057141257	1.031212	0.05892475	181	*Ngp*	0.088359734	1.65211	0.14598
132	*Rhox12*	0.057606898	0.5425635	0.0312554	182	*Tulp1*	0.089171208	0.175255	0.0156277
133	*Bpifb6*	0.05813836	0.22322525	0.01297795	183	*Lgals2*	0.089355653	1.6734	0.14952775
134	*Bpifb2*	0.058319829	0.51096	0.0297991	184	*Trim40*	0.090338372	0.13776095	0.0124451
135	*Ifi27l2b*	0.058494103	0.372117	0.02176665	185	*Chrna4*	0.090405126	0.6591905	0.0595942
136	*Prss2*	0.058737335	0.7016985	0.0412159	186	*Apoa4*	0.091112043	4.19489	0.382205
137	*Klk1b1*	0.058777767	0.488393	0.02870665	187	*Epx*	0.092492564	1.013655	0.09375555
138	*Cilp*	0.059185779	1.747375	0.10341975	188	*Pkd2l1*	0.092768054	0.1570255	0.01456695
139	*Ckm*	0.059744086	0.415257	0.02480915	189	*Tpsb2*	0.092860211	0.9371985	0.08702845
140	*Ppy*	0.0634102	0.9972055	0.063233	190	*Prss37*	0.093316278	0.2843625	0.02653565
141	*Sftpa1*	0.063863747	0.250358	0.0159888	191	*2610028E06Rik*	0.095010662	0.5230955	0.04969965
142	*Pde4c*	0.064993426	0.2255105	0.0146567	192	*Tac2*	0.095255729	3.80501	0.362449
143	*Bhmt*	0.065187102	0.1445493	0.00942275	193	*Sbpl*	0.095929238	3.229985	0.30985
144	*Lypd8*	0.065824782	5.055125	0.3327525	194	*Slc12a3*	0.09607939	0.330395	0.03174415
145	*Khdc1a*	0.066088861	0.498149	0.0329221	195	*Prg3*	0.096986622	1.736755	0.168442
146	*Oas1f*	0.066771672	0.93186	0.06222185	196	*Hmgcs2*	0.097002068	0.318603	0.03090515
147	*Dio3os*	0.067226394	0.9410835	0.06326565	197	*Smtnl1*	0.097476874	0.5895465	0.05746715
148	*Umod*	0.067286914	0.4420815	0.0297463	198	*Creb3l3*	0.097513881	0.193796	0.0188978
149	*Sbp*	0.067599114	0.747924	0.050559	199	*Saa3*	0.100508671	20.54375	2.064825
150	*Cd207*	0.067670896	0.5514845	0.03731945	200	*Myoc*	0.100673075	0.795379	0.08007325

Twelve Aire-dependent TSAs are marked with yellow highlighting.

Supplementary Table 2 │ The list of 57 introns preferentially expressed in *Jmjd6*$^{-/-}$ thymic stroma under the RANKL-stimulated condition

list number	Gene	Intron	The ratio of IR value of Jmjd6-/- sample to that of WT sample	Intron retention value in WT sample	Intron retention value in Jmjd6-/- sample	list number	Gene	Intron	The ratio of IR value of Jmjd6-/- sample to that of WT sample	Intron retention value in WT sample	Intron retention value in Jmjd6-/- sample
1	*S100pbp*	3	3.42825734	0.27047233	0.927248752	30	*Txnl4a*	3	1.706000472	0.082061481	0.139996926
2	*Cbr1*	1	3.30738527	0.04049109	0.133919633	31	*Nop56*	6	1.701135181	0.133692333	0.22742873
3	*Vps11*	10	2.884879665	0.105458228	0.304234297	32	*Man2c1*	16	1.690245267	0.329898845	0.557609962
4	*Prrc2a*	8	2.711950449	0.059476704	0.161297874	33	*Tspan31*	5	1.685067695	0.187475271	0.315908522
5	*Ddx26b*	1	2.685030587	0.081618597	0.21914843	34	*Timm21*	4	1.683695194	0.256928351	0.43258903
6	*Ube2m*	2	2.659745577	0.048185162	0.128160271	35	*Hnrnpl*	7	1.68242653	0.092002312	0.154787131
7	*Hmg20b*	7	2.617445318	0.051739902	0.135426365	36	*Chd9*	2	1.67849814	0.985270891	1.653775357
8	*Prrc2a*	30	2.391340405	0.081325095	0.194475985	37	*Fuz*	8	1.647475947	0.782850521	1.289727404
9	*Plekhj1*	2	2.387277122	0.226751957	0.54131976	38	*Plekhj1*	5	1.63745942	0.341541693	0.559260663
10	*Pkhd1l1*	75	2.334304807	0.549947445	1.283744964	39	*Irf3*	5	1.630857889	0.32267447	0.526236204
11	*Cops7a*	8	2.307608184	0.071846992	0.165794708	40	*Rbm5*	23	1.628831922	0.159769168	0.26023712
12	*Srsf10*	2	2.238861496	0.165780036	0.37115854	41	*Uba7*	6	1.609692625	0.307689811	0.49528602
13	*Skint8*	1	2.175970102	0.129118081	0.280957084	42	*Kidins220*	29	1.599859191	0.138414848	0.221444267
14	*Ap1m2*	5	2.170550377	0.088066785	0.191153394	43	BC029214	1	1.599555002	0.689095368	1.102245943
15	*Eml3*	17	2.150250987	0.218042424	0.468845937	44	*Irf7*	5	1.597539383	0.119085713	0.190244117
16	*Atp6v0b*	4	2.085221058	0.075801131	0.158062115	45	*Naprt1*	1	1.58910856	0.25337335	0.402637759
17	*Taf6l*	14	1.897699752	0.148860727	0.282492965	46	*Snrnp48*	6	1.574525159	0.35379915	0.557065663
18	*Scarf2*	7	1.887998528	0.172947711	0.326525023	47	*Srpk2*	8	1.572642575	0.096223463	0.151325114
19	*H2-Ke6*	2	1.880672697	0.097040036	0.182500547	48	*Plekhj1*	4	1.57071895	0.418674079	0.65761931
20	*Aire*	2	1.867887148	0.263046914	0.49134195	49	*Snapin*	1	1.559616974	0.113680697	0.177298345
21	*Zmym5*	3	1.849485249	0.163334148	0.302084097	50	*Akap8*	8	1.55456425	0.196468177	0.305422404
22	*Chd3*	37	1.845131443	0.311798592	0.575309386	51	AI314180	33	1.548912041	0.074812968	0.115878706
23	*Hes6*	3	1.839043869	0.061730213	0.11352457	52	*Aire*	10	1.52640764	0.292625432	0.446665694
24	*Araf*	5	1.814846341	0.144023705	0.261380894	53	*Zfp692*	7	1.523996414	0.347554975	0.529672535
25	0610011F06Rik	5	1.809018104	0.414154686	0.749213325	54	*Tmc3*	13	1.512615164	0.25612313	0.38741573
26	*Elmo3*	5	1.806219078	0.322610213	0.582704721	55	*Coq2*	2	1.505363313	0.183380669	0.276054532
27	*Actr1b*	5	1.773556426	0.175335923	0.310968153	56	*Samd4b*	12	1.503608672	0.529322792	0.795894341
28	*Per3*	20	1.740305714	0.314014268	0.546480824	57	*Ikbkb*	12	1.502055826	0.220970325	0.331909763
29	*Prrc2a*	9	1.711886285	0.117783468	0.201631903						

Intron retention (IR) value was calculated by dividing intronic FPKM value by conventional FPKM value for each gene.
